# “There’s an app for that” — A novel tool to help community correction populations learn strategies to decrease HIV risk behaviors after release

**DOI:** 10.1186/1940-0640-10-S1-A15

**Published:** 2015-02-20

**Authors:** Julie Gray, Jennifer Pankow, Wayne EK Lehman, Grace Rowan, Kevin Knight

**Affiliations:** 1 Institute of Behavioral Research, Texas Christian University, Fort Worth, TX, 76129, USA

## 

During the first 5 years of the Disease Risk Reduction Project, an in-prison intervention curriculum was developed and tested that focused on decreasing risky sexual and drug use behaviors after release. The *WaySafe* intervention curriculum was delivered during the 2 months prior to release from incarceration. Compared to the control group, those in *WaySafe* showed greater gains on all composite measures (e.g., *HIV knowledge confidence*, *avoidance of risky sex*, *avoidance of risky drug use*, *HIV testing awareness*, *and risk reduction skills*. In addition, *WaySafe* was followed by a take-home workbook to be completed post-release. The goal of the take-home assignment was to reinforce skills and strategies learned through *WaySafe* during the offenders’ transition back into the community.

With lessons learned from the take-home assignment, as an important next step, the current project is focusing on adapting and delivering the intervention curriculum in community correction populations. Because computerized interventions have demonstrated promising results with substance abuse treatment [1], the new *StaySafe* intervention is formatted as an engaging computer-driven program. Computer applications in these treatment setting studies were found to be similarly or more efficacious than traditional counselor-led treatment sessions [1]. These findings suggest adoption of new technologies to deliver treatment. *StaySafe* will incorporate the same evidence-based cognitive principles as *WaySafe* in 12 self-paced modules that can be self-administered during the first 6 months under community supervision. This presentation will provide an overview of the computerized *StaySafe* approach and discuss the technological development of *StaySafe* elements. We will demonstrate the use of computerized, interactive, cognitive mapping strategies for presentation of ideas within the program as well as strategies for eliciting participant input and responses, including strengths and weaknesses of different response strategies (e.g., recording audio responses, using a keyboard or touchscreen to type responses, writing responses on the touchscreen, or choosing a response from a predetermined, pull-down list). Examples of several *StaySafe* computerized elements will be demonstrated.

## Trial registration

Clinicaltrials.gov NCT01900210
